# Significance of glycolytic metabolism-related protein expression in colorectal cancer, lymph node and hepatic metastasis

**DOI:** 10.1186/s12885-016-2566-9

**Published:** 2016-07-26

**Authors:** Sandra Fernandes Martins, Ricardo Amorim, Marta Viana-Pereira, Céline Pinheiro, Ricardo Filipe Alves Costa, Patrícia Silva, Carla Couto, Sara Alves, Sara Fernandes, Sónia Vilaça, Joaquim Falcão, Herlander Marques, Fernando Pardal, Mesquita Rodrigues, Ana Preto, Rui Manuel Reis, Adhemar Longatto-Filho, Fátima Baltazar

**Affiliations:** 1 Life and Health Sciences Research Institute (ICVS), School of Health Sciences, University of Minho, Campus de Gualtar, 4710-057 Braga, Portugal; 2 ICVS/3B’s - PT Government Associate Laboratory, Braga/Guimarães, Portugal; 3 Surgery Department, Hospitalar Center Trás-os-Montes e Alto Douro, Chaves Unit, Chaves, Portugal; 4 Molecular Oncology Research Center, Barretos Cancer Hospital, Barretos, São Paulo Brazil; 5 Barretos School of Health Sciences Dr. Paulo Prata - FACISB, Barretos, São Paulo Brazil; 6 General Surgery Resident at Braga Hospital, Braga, Portugal; 7 Hepatobiliary Unit, Braga Hospital, Braga, Portugal; 8 Oncology Department, Braga Hospital, Braga, Portugal; 9 Pathology Department, Braga Hospital, Braga, Portugal; 10 Coloproctology Unit, Braga Hospital, Braga, Portugal; 11 Center of Molecular and Environmental Biology (CBMA)/Department of Biology, University of Minho, Braga, Portugal; 12 Laboratory of Medical Investigation (LIM) 14, Faculty of Medicine, University of São Paulo, São Paulo, Brazil

**Keywords:** Colorectal cancer, Lymph node metastasis, Hepatic metastasis, Monocarboxylate transporters, CD147, GLUT1

## Abstract

**Background:**

Colorectal cancer (CRC) is one of the most common malignancies and a leading cause of cancer death worldwide. Most cancer cells display high rates of glycolysis with production of lactic acid, which is then exported to the microenvironment by monocarboxylate transporters (MCTs). The main aim of this study was to evaluate the significance of MCT expression in a comprehensive series of primary CRC cases, lymph node and hepatic metastasis.

**Methods:**

Expressions of MCT1, MCT4, CD147 and GLUT1 were studied in human samples of CRC, lymph node and hepatic metastasis, by immunohistochemistry.

**Results:**

All proteins were overexpressed in primary CRC, lymph node and hepatic metastasis, when compared with non-neoplastic tissue, with exception of MCT1 in lymph node and hepatic metastasis. MCT1 and MCT4 expressions were associated with CD147 and GLUT1 in primary CRC. These markers were associated with clinical pathological features, reflecting the putative role of these metabolism-related proteins in the CRC setting.

**Conclusion:**

These findings provide additional evidence for the pivotal role of MCTs in CRC maintenance and progression, and support the use of MCTs as biomarkers and potential therapeutic targets in primary and metastatic CRC.

## Background

Colorectal cancer (CRC) is the third most common cancer in men and the second in women, being one of the most prevalent diseases of the occidental world [1].

Altered metabolism in cancer cells was recently recognized as a hallmark of cancer [2]. Most cancer cells display high rates of glycolysis with production of lactic acid, which is then exported to the microenvironment, leading to a decrease in extracellular pH. High levels of lactate and low pH has been associated with increased malignant features, including cell invasion [3], suppression of immune response [4] tumour proliferation, angiogenesis and metastasis [5, 6]. Extracellular lactate has been associated with poor prognosis in cancer [6, 7] and monocarboxylate transporters (MCTs) are essential players in the maintenance of the glycolytic metabolism being both lactate transporters and pH regulators [8–11]. MCTs are currently seen as promising therapeutic targets in cancer, with encouraging results in vitro and in vivo models [12–21].

The MCT family comprises 14 members; however, only the first four (MCT1-4) were identified as mediating the proton-coupled transport of monocarboxylic acids across the plasma membrane [22–24]. It is currently believed that the MCT isoform 4 mediates mostly lactate efflux, whereas MCT1 performs the uptake of lactate that is used by oxidative cancer cells [17, 25, 26]. CD147 is co-expressed with MCT1 and MCT4 for proper plasma membrane expression and catalytic activity [27–30].

Data on the role of MCTs in CRC is somewhat contradictory. Koukoukaris et al. [31] described MCT1 and MCT2 expression in cancer cells and tumour-associated fibroblasts, with weak MCT4 expression in the tumour stroma. On the other hand, our group described higher MCT1 and MCT4 CRC membrane expression and lower of MCT2 expression, comparing with the adjacent normal tissue [32]. However, despite these controversies, positive MCT4 expression in CRC has been associated with poor prognosis [33, 34], supporting the role of this MCT isoform in CRC malignancy. Interestingly, the expression of MCT1 and MCT4 is described to vary along tumor progression, especially for MCT1. There are reports showing decrease in MCT1 expression during transition from normality to malignancy in the colonic mucosa [35, 36]. However, upregulation of MCT1 has also been described in advanced CRC tumors [31, 32]. Besides MCTs, lactate can be also transported by sodium-coupled monocarboxylate co-transporters (SMCTs), which are expressed in the apical membrane of colon [37–39]. However, SMCT1 expression is frequently silenced in aberrant colon precursor lesions and cancer [40, 41].

The aim of the present study was to evaluate the role of MCTs in CRC, by assessing the immunohistochemical expression of the MCT isoforms 1, 4, CD147 and the glycolytic metabolic marker GLUT1, and correlate their expressions with clinicopathological parameters in a comprehensive CRC series, including primary tumours and both lymph node and hepatic metastasis. Our results provide additional evidence of MCTs role in primary CRC and CRC metastasis, supporting their use as biomarkers and potential therapeutic targets in primary and metastatic CRC.

## Methods

### CRC primary tumour and metastasis human samples

Tissue samples and data from 487 patients treated in Hospital de Braga, Portugal, between 1st January of 2005 and 1st January of 2010 with CRC diagnosis were collected prospectively. Tumour localization was recorded and classified as colon and rectum (between anal verge and 15 cm at rigid rectoscopy). The histological type of CRC was classified by an experienced pathologist and tumour staging was graded according to the TNM classification, sixth edition [42]. Tissue samples of CRC lymph node metastasis were selected from the previous series, comprising 210 patients.

Additionally, an independent series of 45 patients with histological diagnosis of CRC hepatic metastasis operated between 1st January of 2003 and 1st January of 2011 was retrieved from the files of Hospital de Braga and data were retrospectively collected.

CRC samples and CRC lymph node metastasis were included into tissue microarrays (TMAs). Prior to TMA construction, haematoxylin and eosin sections were reviewed to select representative areas of the tumour. Normal-adjacent tissue was also included in the TMAs for primary tumours. Each case was represented in the TMA by at least two cores of 0.6 mm.

The study protocol was approved by the Ethics Committee of Hospital de Braga. The data of CRC and lymph node metastasis series were collected prospectively, patients were informed and signed a written consensus for collecting data and samples collection.

### Immunohistochemistry

Protein expression in primary CRC samples, lymph nodes and hepatic metastasis was evaluated by immunohistochemistry, as previously described [43]. Detailed information is depicted in Table 1. The specificity of MCT1 and MCT4 antibodies has been demonstrated in previous publications [19–21].

**Table 1:** Detailed aspects of the immunocytochemical and immunohistochemical procedure used to visualize the different proteins

Protein	Antigen retrieval	Positive Control	Peroxidase inactivation	Detection system	Antibody		
					Company	Dilution	Incubation period
MCT1	Citrate buffer (10 mM, pH = 6.0) 98 °C; 20 min.	Colon carcinoma	0.3 % H_2_O_2_ in methanol, 30 min.	R.T.U. VECTASTAIN® Elite_®_ ABC Kit (Vector Laboratories)	Chemicon Ref. AB3538P	1:300	Overnight
MCT4	Citrate buffer (10 mM, pH = 6.0) 98 °C; 20 min.	Colon carcinoma	3 % H_2_O_2_ in methanol, 30 min.	Ultravision Detection System Anti-polyvalent, HRP (Lab Vision Corporation)	Santa Cruz Biotechnology Ref. sc-50329	1:200	2 h
CD147	EDTA (1 mM, pH = 8) 98 °C; 15 min.	Colon carcinoma	3 % H_2_O_2_ in methanol, 10 min.	Ultravision Detection System Anti-polyvalent, HRP (Lab Vision Corporation)	Zymed Ref. 18-7344	1:500	2 h
GLUT1	Citrate buffer (10 mM, pH = 6.0) 98 °C; 10 min.	Skin	3 % H_2_O_2_ in methanol, 10 min.	Ultravision Detection System Anti-polyvalent, HRP (Lab Vision Corporation)	Abcam Ref. ab15309-500	1:500	2 h

### Immunohistochemical evaluation

Immunohistochemical evaluation was performed as previously described [32].

Briefly, sections were scored semi-quantitatively for immunoreaction extension (score 0–3) and intensity (score 0–3). Immunoreaction final score was defined as the sum of both parameters, and grouped as negative (0–2) and positive (≥3). Both cytoplasm and plasma membrane staining were assessed, but for statistical analysis only membrane staining was considered. Evaluation of protein expressions was performed by blind analysis by two observers and discordant cases were discussed in a double-head microscope in order to define the final score.

### KRAS and BRAF mutation screening

Mutation analysis of BRAF (exon 15) and KRAS (codons 12 and 13) hotspot mutations, was performed by PCR, using primers and methods previously described [44, 45], followed by direct sequencing.

### Microsatellite Instability analysis

Microsatellite Instability (MSI) was determined using a multiplex PCR of five quasimonomorphic mononucleotide repeat markers was end-labeled with a fluorescent dye (NR27, NR21, NR24, BAT25 and BAT26), as described [46]. PCR was performed using the Qiagen Multiplex PCR Kit, and products were separated using the ABI 3730 XL capillary genetic analyzer (Applied Biosystems) and analyzed using the GeneMapper 4.1 software (Applied Biosystems). Cases exhibiting instability at three or more markers were considered as having high MSI (MSI-H), those with instability at one or two markers being defined as having low MSI (MSI-L), and those showing no instability were defined as microsatellite stable (MSS), as described [47].

### Statistical analysis

All data were analyzed using the Statistical Package for the Social Sciences, version 19.0 (SPSS Inc., Chicago, Illinois, USA). Comparisons were examined for statistical significance using Pearson’s chi-square (*χ*2) test and Fisher’s exact test (when *n <* 5).

Expression differences between lymph node metastasis and primary CRC were tested with McNemar test. Survival curves were determined for overall survival by the Kaplan–Meier method using log-rank test.

Predictive factors of prognosis were identified by means of Cox proportional hazards regression models, which were used to estimate hazard ratios (HR) and their 95 % confidence intervals in univariate and multivariate analysis. For multivariate analysis, variables that reached a *p* value <0.1 at univariate analysis were included. The threshold for significant p values was established as *p ≤* 0.05.

## Results

### MCT4, CD147 and GLUT1 are overexpressed in CRC primary tumours, lymph node and hepatic metastasis

**Fig. 1:**
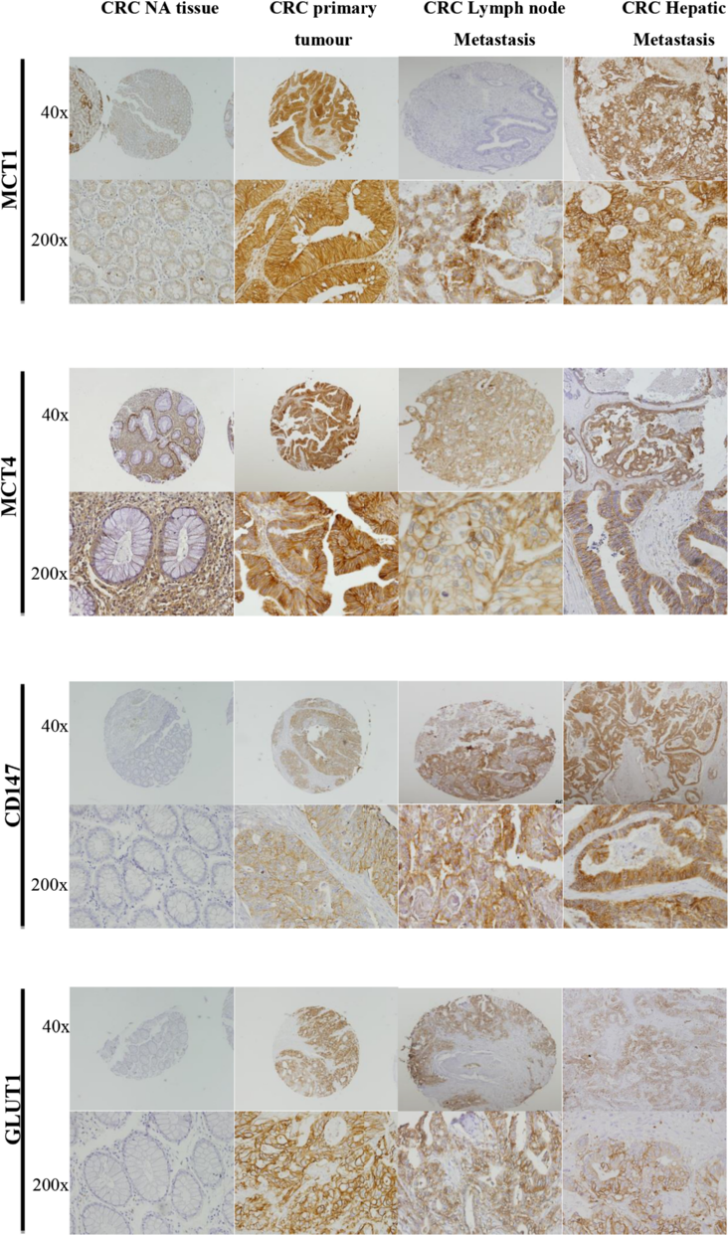
Representative immunohistochemical expression of proteins in CRC NA tissue, CRC primary tumour, CRC lymph node metastasis and CRC hepatic metastasis. Representative immunohistochemical expression of MCT1, MCT4, CD147 and GLUT1 in CRC NA tissue, CRC primary tumour and CRC lymph node metastasis and CRC hepatic metastasis. (40x and 200x magnification)

To infer about the importance of the proteins MCT1, MCT4, CD147 and GLUT1 in the progression of CRC, their expression was evaluated by immunohistochemistry in 487 samples of CRC, 210 samples of CRC lymph node metastasis and 45 samples of hepatic metastasis. Representative images of MCT1, MCT4, CD147 and GLUT1 positive staining in CRC normal adjacent (NA) epithelium, primary tumour, lymph node and hepatic metastasis are presented in Fig. 1.

**Fig. 2:**
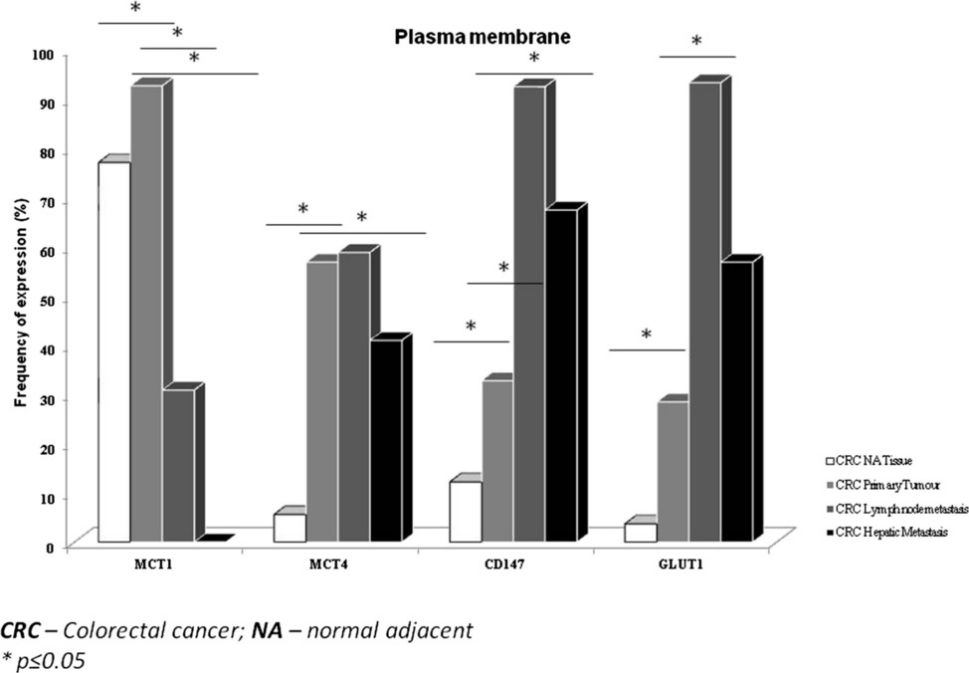
Frequency of protein staining in CRC NA tissue, CRC primary tumour and CRC lymph node and hepatic metastasis. Frequency of MCT1, MCT4, CD147 and GLUT1 plasma membrane staining in CRC NA (normal adjacent) tissue, CRC primary tumour and CRC lymph node and hepatic metastasis. *p ≤ 0.05

All proteins were overexpressed at the plasma membrane of primary CRC tumours, CRC lymph node metastasis and CRC hepatic metastasis when compared with CRC NA tissue (*p* < 0.001, Fig. 2), with exception for MCT1 in CRC lymph node and hepatic metastasis. We detected a significant increase in both MCT1 and MCT4 expressions in CRC primary tumour (*p* < 0.001, for both), with a decrease of MCT1 expression in CRC primary tumour to lymph node and hepatic metastasis (*p* < 0.001, for both) and a decrease of MCT4 expression in CRC primary tumour to hepatic metastasis (*p* = 0.0001). Compared to the MCTs expressions, the percentage of CD147 and GLUT1 positivity reactions were lower in CRC primary tumour; however, there was an increase in their expression from CRC primary tumour to lymph node (*p* < 0.001 and *p* = 0.003, respectively) and hepatic metastasis (*p* < 0.001, for both) (Fig. 2). In the context of another study (yet unpublished), we analyzed 45 samples of non-neoplastic lymph nodes where we saw that all cases were negative for MCT1, MCT2, MCT4 and CD147 and only one case was positive for GLUT1 (2.2 %).

We also matched the expression of these metabolism-related proteins in CRC hepatic metastasis with NA hepatic tissue, and we observed that these proteins presented a low expression in the liver tissue (*p* < 0.001, for all proteins, data not shown), namely MCT4 and GLUT1 with no expression and MCT1 and CD147 with 64.4 and 30 %, respectively, at NA hepatic tissue.

Since CRC primary tumours and lymph node metastasis belong to the same group of patients, we could compare the expression of the proteins in the two types of samples. We observed that MCT1, CD147 and GLUT1 positivity in CRC primary tumour samples associates with MCT1, CD147 and GLUT1 positivity in their respective lymph node metastasis (*p* < 0.001, *p* < 0.001 and *p* = 0.003 respectively). On the other hand, MCT4 expression in lymph node metastasis seems to be independent of its expression in CRC primary tumour. Interestingly, primary CRC with negative MCT1 and MCT4 expressions can originate lymph node metastasis with positive expression for both markers. Detailed information is depicted in Table 2.

**Table 2:** Assessment of associations between protein plasma membrane expression in CRC primary tumour and in CRC lymph node metastasis

		LN_MCT1			*p*
MCT1		Negative	Positive	Total	0.000
		(%)	(%)		
CRC_MCT1	Negative (%)	80 % (*n* = 8)	20,0 % (*n* = 2)	100 % (*n* = 10)	
	Positive (%)	69.5 % (*n* = 73)	30.5 % (*n* = 32)	100 % (*n* = 105)	
	Total	70.4 % (*n* = 81)	29.6 % (*n* = 34)	100 % (*n* = 115)	
MCT4		LN_MCT4			*p*
		Negative (%)	Positive (%)	Total	0.568
CRC_MCT4	Negative (%)	45.0 % (*n* = 18)	55.0 % (*n* = 22)	100 % (n = 40)	
	Positive (%)	40.3 % (*n* = 27)	59.7 % (*n* = = 40)	100 % (*n* = 67)	
	Total	100 % (*n* = 45)	100 % (*n* = 62)	100 % (*n* = 107)	
CD147		LN_CD147			*p*
		Negative (%)	Positive (%)	Total	0.000
CRC_CD147	Negative (%)	25.3 % (*n* = 20)	74.7 % (*n* = 59)	100.0 % (*n* = 79)	
	Positive (%)	14.7 % (*n* = 5)	85.3 % (*n* = 29)	100.0 % (*n* = 34)	
	Total	22.1 % (*n* = 25)	77.9 % (*n* = 88)	100.0 % (*n* = 113)	
GLUT1		LN_GLUT1			*p*
		Negative (%)	Positive (%)	Total	0.003
CRC_GLUT1	Negative (%)	55.6 % (*n* = 35)	44.4 % (*n* = 28)	100.0 % (*n* = 63)	
	Positive (%)	26.5 % (*n* = 9)	73.5 % (*n* = 25)	100.0 % (*n* = 34)	
	Total	45.4 % (*n* = 44)	54.6 % (*n* = 53)	100.0 % (*n* = 97)	

*CRC* Colorectal cancer, *LN* Lymph node

### MCT1 and MCT4 expression is associated with CD147 and GLUT1 in CRC primary tumour and in lymph node and hepatic metastasis

To better characterize the role of MCT1 and MCT4 in our samples, we assessed the association with their chaperone CD147 and the glycolytic marker GLUT1. MCT1 expression was associated with CD147 (*p* = 0.003) in CRC primary tumour samples and with GLUT1 in CRC hepatic metastasis (*p* = 0.002) (Table 3). The expression of MCT4 was associated with GLUT1 (*p* = 0.001) in CRC primary tumour and with CD147 expression (*p* = 0.050) (Table 3). MCT4 positivity was also associated with CD147 and GLUT1 in CRC lymph node metastasis samples (*p* = 0.007 and *p* = 0.019, respectively) and hepatic metastasis samples (*p* = 0.019 and *p* < 0.001, respectively) (Table 3).

**Table 3:** Assessment of associations between MCTs and CD147/GLUT1 in CRC primary tumour and in CRC primary tumour and metastasis

CRC primary tumour	CD147			GLUT1		
	*n*	Positive (%)	*p*	*n*	Positive (%)	*p*
MCT1						
Positive	452	157 (34.7 %)	0.003	425	126 (29.6 %)	0.076
Negative	36	4 (11.1 %)		33	5 (15.2 %)	
MCT4						
Positive	269	100 (37.2 %)	0.050	262	90 (34.4 %)	0.001
Negative	203	58 (28.6 %)		191	38 (19.9 %)	
CRC lymph node metastasis						
MCT1						
Positive	31	30 (96.8 %)	0.100	28	24 (85.7 %)	0.165
Negative	66	56 (84.8 %)		44	31 (70.5 %)	
MCT4						
Positive	56	54 (96.4 %)	0.007	46	39 (84.8 %)	0.019
Negative	39	30 (76.9 %)		25	15 (60.0 %)	
CRC hepatic metastasis						
MCT1						
Positive	33	24 (72.7 %)	0.097	33	23 (69.7 %)	0.002
Negative	8	3 (37.5 %)		9	1 (11.1 %)	
MCT4						
Positive	18	16 (88.9 %)	0.019	18	18 (100 %)	<0.001
Negative	25	13 (52.0 %)		25	6 (24.0 %)	

*CRC* Colorectal cancer

### MCT1, MCT4, CD147 and GLUT1 expressions are associated with poor prognostic features

In order to assess the clinicopathological value of the expression of MCTs, CD147 and GLUT1, we sought for associations with the clinicopathological data of CRC primary tumours. The following associations were found: positive association between MCT1 expression and older patients (*p* = 0.007, Table 4); CD147 positivity and bigger tumours and higher tumour penetration (*p* = 0.003, *p* = 0.034 Table 5); and GLUT1 with exophytic macroscopic appearance and low CEA levels (*p* = 0.023 and *p* = 0.050 respectively, Table 4), poorly differentiated tumours *(p* = 0.009, Table 5) and a trend to associate with the presence of lymph node metastasis *(p* = 0.058, Table 5). No significant correlations were found among MCTs, CD147 and GLUT1 and the molecular markers KRAS or BRAF mutations and Microsatellite Instability status.

**Table 4:** Assessment of associations between proteins plasma membrane expression and clinical data in CRC primary tumours

	MCT1			MCT4			CD147			GLUT1		
	*n*	Positive (%)	*p*	*n*	Positive (%)	*p*	*n*	Positive (%)	*p*	*n*	Positive (%)	*p*
Sex												
Male	314	92.7	0.934	302	57.3	0.801	312	31.4	0.391	294	28.6	0.969
Female	186	92.5		180	56.1		182	35.2		169	28.4	
Age (years)												
≤45	23	78.3	0.007	21	47.6	0.383	23	21.7	0.247	23	26.1	0.792
>45	477	93.3		461	57.3		471	33.3		440	28.6	
Presentation												
Asymptomatic	87	93.1	0.844	84	48.8	0.102	87	36.8	0.383	83	28.9	0.928
Symptomatic	413	92.5		398	58.5		407	31.9		380	28.4	
Localization												
Colon	360	92.5	0,891	351	59.3	0.080	359	33.4	0.625	338	29.3	0.541
Rectum	140	92.9		131	50.4		135	31.1		125	26.4	
Macroscopic Appearence												
Polypoid	254	92.9	0.492	247	54.7	0.245	249	33.3	0.798	239	23.8	0.023
Ulcerative	116	91.4		115	54.8		118	32.3		111	29.7	
Infiltrative	42	85.7		40	62.5		40	27.5		35	25.7	
Exophytic	42	95.2		37	70.3		41	29.3		34	50.0	
Vilosous	2	100		2	100		2	0.0		2	0.0	
CEA (ng/mL)												
<5	122	90.2	0.568	115	60.0	0.665	118	33.1	0.455	111	36.9	0.05
≥5	272	91.9		269	57.6		270	29.3		256	22.7

**Table 5:** Assessment of associations between proteins plasma membrane expression and pathological data in CRC primary tumours

	MCT1			MCT4			CD147			GLUT1		
	*n*	Positive (%)	*p*	*n*	Positive (%)	*p*	*n*	Positive (%)	*p*	*n*	Positive (%)	*p*
Tumor size (cm)												
≤4.5	286	93.4	0.389	278	54.7	0.265	283	27.9	0.003	267	29.6	0.466
>4.5	182	91.2		175	60.0		180	41.1		167	26.3	
Histological Type												
Adenocarcinoma	417	92.8	0.456	402	57.0	0.862	411	33.6	0.787	386	28.2	0.389
A. Mucinous	51	90.2		49	57.1		52	28.8		46	26.1	
A. Invasive	24	95.8		24	54.2		23	26.1		23	39.1	
Signet ring and mucinous	4	75.0		3	33.3		4	25.0		4	0.0	
Differentiation												
Well-differentiated	219	93.2	0.271	213	56.8	0.070	217	34.6	0.875	202	21.3	0.009
Moderately-differentiated	209	93.3		204	55.4		206	32.5		197	35.0	
Poorly-differentiated	49	85.7		43	69.8		48	29.2		43	39.5	
Undifferentiated	4	100.0		3	0.0		4	25.0		3	33.3	
Tumour Penetration												
Tis	5	100.0	0.946	6	16.7	0.277	4	25.0	0.034	5	0.0	0.436
T1	30	90.0		28	50.0		30	13.3		27	29.6	
T2	59	93.2		58	56.9		59	30.5		55	21.8	
T3	376	92.6		359	57.7		371	33.2		350	29.4	
T4	24	91.7		25	64.0		24	54.2		20	35.0	
Spread to lymph nodes												
Absent	280	92.5	0.888	272	54.0	0.269	277	32.5	0.876	263	25.5	0.058
Present	204	92.2		196	59.2		202	33.2		187	33.7	
Vessel invasion												
Absent	159	94.3	0.255	159	58.5	0.541	156	33.3	0.817	150	25.3	0.194
Present	314	91.4		299	55.5		313	32.3		291	31.3	
TNM												
Stage I	77	92.1	0.566	77	52.0	0.464	77	22.1	0.147	74	23.3	0.206
Stage II	183	92.9		179	57.0		181	36.5		173	26.0	
Stage III	155	94.2		151	57.6		154	34.4		142	30.3	
Stage IV	75	88.0		67	59.7		73	31.5		66	39.4	
BRAF mutations												
Negative	87	94.3	1.000	56	65.9	0.608	33	38.4	0.641	16	19.8	0.196
Positive (V600E)	4	100		2	50.0		2	50.0		2	50.0	
KRAS mutations (codon12/13 and 61)												
Negative	78	96.3	0.437	51	64.6	0.217	27	34.2	0.668	17	21.8	0.411
Positive	41	93.2		31	75.6		16	38.1		6	15.4	
Microsatellite Instability												
Negative	102	95.3	0.986	66	65.3	0.335	38	36.5	0.321	20	20.2	0.984
Positive (MSI-L + MSI-H)	20	95.2		16	76.2		5	25.0		4	20.0	

Assessment of associations between plasma membrane protein expression in lymph node metastasis and clinicopathological data of CRC primary tumour revealed a significant association between MCT4 and tumours localized in colon (colon cancer *(p* = 0.032, Table 6) and tumour penetration (*p* = 0.034, Table 7), and for CD147 positivity and tumour differentiation (*p* = 0.033, Table 7).

**Table 6:** Assessment of associations between proteins plasma membrane expression in CRC lymph node metastasis and clinical data

	MCT1			MCT4			CD147			GLUT1		
	*n*	Positive (%)	*p*	*n*	Positive (%)	*p*	*n*	Positive (%)	*p*	*n*	Positive (%)	*p*
Sex												
Male	77	25 (32.5)	0.581	74	46 (62.2)	0.317	77	62 (91.9)	0.159	71	47 (76.7)	0.523^a^
Female	40	11 (27.5)		40	21 (52.5)		40	34 (82.4)		38	22 (86.4)	
Age (years)												
≤45	10	3 (30.0)	1.000^a^	8	4 (50.0)	0.715^a^	9	8 (75.0)	0.228^a^	8	5 (40.0)	0.053^a^
>45	107	33 (30.8)		106	63 (59.4)		108	88 (89.8)		101	64 (82.8)	
Presentation												
Asymptomatic	19	6 (31.6)	0.933	18	8 (44.4)	0.178	22	18 (88.9)	1.000^a^	16	10 (60.0)	0.109^a^
Symptomatic	98	30 (30.6)		96	59 (61.5)		95	78 (88.5)		93	59 (83.1)	
Localization												
Colon	94	28 (29.8)	0.642	91	58 (63.7)	0.032	95	81 (88.9)	0.681^a^	88	57 (80.7)	0.698^a^
Rectum	23	8 (34.8)		23	9 (39.1)		22	15 (86.7)		21	12 (75.0)	
Macroscopic Appearence												
Polypoid	47	14 (29.8)	0.596	47	27 (57.4)	0.534	45	36 (86.1)	0.701	45	25 (84.0)	0.500
Ulcerative	31	7 (22.6)		30	20 (66.7)		34	28 (85.7)		28	20 (70.0)	
Infiltrative	13	5 (38.5)		13	6 (46.2)		12	11 (90.9)		11	6 (100.0)	
Exophytic	14	6 (42.9)		13	7 (53.8)		14	12 (100.0)		14	11 (81.8)	
Vilosous	1	0 (0.0)		1	0 (0.0)		1	1 (100.0)		1	1 (100.0)	
CEA (ng/mL)												
<5	71	21 (29.6)	0.354	67	42 (62.7)	0.434	68	56 (91.1)	0.120	65	40 (85.0)	0.237^a^
≥5	25	5 (20.0)		26	14 (53.8)		26	23 (78.3)		23	13 (69.2)	

^a^Comparisons were examined for statistical significance using Fisher’s exact test (when *n* < 5)

**Table 7:** Assessment of associations between proteins plasma membrane expression in CRC lymph node metastasis and pathological data

	MCT1		MCT4				CD147			GLUT1		
	*n*	Positive (%)	*p*	*n*	Positive (%)	*p*	*n*	Positive (%)	*p*	*n*	Positive (%)	*p*
Tumor size (cm)												
≤4.5	67	26 (38.8)	0.065	65	38 (58.5)	0.692	68	53 (92.5)	0.492^a^	65	43 (76.7)	0.548^a^
>4.5	45	10 (22.2)		45	28 (62.2)		45	40 (87.5)		40	25 (84.0)	
Histological Type												
Adenocarcinoma	92	32 (34.8)	0.287	92	54 (58.7)	0.376	90	76 (88.2)	0.826^a^	85	58 (77.6)	0.084^a^
A. Mucinous	16	2 (12.5)		15	7 (46.7)		18	14 (85.7)		17	6 (100.0)	
A. Invasive	6	1 (16.7)		6	5 (83.3)		6	5 (100.0)		6	4 (100.0)	
Signet ring and mucinous	3	1 (33.3)		1	1 (100.0)		3	1 (100.0)		1	1 (0.0)	
Differentiation												
Well-differentiated	41	18 (43.9)	0.152	40	23 (57.5)	0.493	41	36 (91.7)	0.033^a^	38	26 (76.9)	0.902^a^
Moderately-differentiated	51	13 (25.5)		50	28 (56.0)		50	43 (86.0)		47	29 (79.3)	
Poorly-differentiated	23	5 (21.7)		22	15 (68.2)		23	16 (93.8)		22	13 (84.6)	
Undifferentiated	1	0 (0.0)		1	0 (0.0)		2	1 (0.0)		1	1 (100.0)	
Tumour Penetration												
T1	2	0 (0.0)	0.408	1	0 (0.0)	0.034	2	1 (100.0)	0.665^a^	1	1 (100.0)	0.653^a^
T2	5	2 (40.0)		4	3 (75.0)		4	3 (100.0)		4	3 (66.7)	
T3	101	22 (32.7)		99	62 (62.6)		101	83 (89.2)		96	61 (78.7)	
T4	9	1 (11.1)		10	2 (20.0)		10	9 (77.8)		8	4 (100.0)	
Spread to lymph nodes												
Absent	9	4 (44.4)	0.450^a^	8	6 (75.0)	0.465^a^	10	8 (87.5)	1.000^a^	8	6 (100.0)	0.326^a^
Present	96	28 (29.2)		94	54 (57.4)		96	77 (89.6)		90	55 (76.4)	
Vessel invasion												
Absent	30	12 (40.0)	0.259	29	20 (69.0)	0.288	33	28 (89.3)	1.000^a^	30	16 (81.3)	1.000^a^
Present	80	23 (28.8)		78	45 (57.7)		79	62 (88.7)		73	49 (81.6)	
TNM												
Stage III	84	28 (33.3)	0.338	82	52 (63.4)	0.107	82	66 (92.4)	0.076	79	48 (81.3)	0.632
Stage IV	33	8 (24.2)		32	15 (46.9)		35	30 (80.0)		30	21 (76.2)	

^a^Comparisons were examined for statistical significance using Fisher’s exact test (when *n* < 5)

In CRC hepatic metastasis, we observed associations between MCT1 and colon tumour localization (*p* = 0.022) (Table 8).

**Table 8:** Assessment of associations between proteins expression in CRC hepatic metastasis and anatomopatological data from primary tumour and clinical data from hepatic metastasis series

Anatomopatological data from Primary tumours	MCT1			MCT4			CD147			GLUT1		
	*n*	Positive (%)	*p*	n	Positive (%)	*p*	*n*	Positive (%)	*p*	n	Positive (%)	*p*
Localization												
Colon	7	42.8	0.022	7	28.6	0.682	7	42.8	0.190	7	42.8	0.443
Rectum	38	86.8		37	43.2		36	72.2		37	59.4	
CRC Stage												
I + II	7	71.4	0.637	8	62.5	0.250	8	75.0	1.000	8	62.5	1.000
III + IV	34	79.4		32	37.5		31	67.7		32	56.2	
Vessel invasion												
Absent	4	50.0	0.681	4	50.0	0.683	4	50.0	0.560	5	80.0	0.346
Present	28	50.0		28	39.3		27	74.1		28	50.0	
Clinical data from Hepatic Metastasis												
Localization												
One hepatic lobe	30	80.0	1.000	30	50.0	0.251	30	73.3	0.129	30	60.0	1.000
Both hepatic lobe	10	80.0		9	22.2		9	44.4		8	62.5	
Size												
≤5 cm	39	76.9	0.316	37	43.2	1.000	37	70.3	0.373	36	58.3	1.000
>5 cm	7	100.0		6	33.3		6	50.0		6	50.0	

**Fig. 3:**
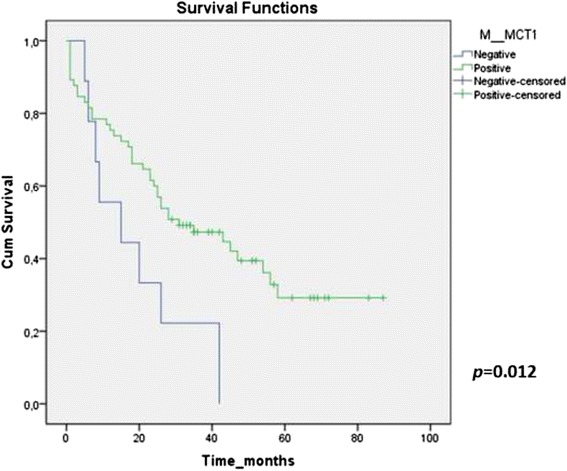
Kaplan-Meyer survival curve of MCT1 plasma membrane expression in CRC. stage IV. The illustration represents the survival curve related to MCT1 plasma membrane expression in CRC stage IV. Patients with negative expression of MCT1 show shorter survival (continuous line), whereas longer survival values were obtained for patients with MCT1 positive expression (interrupted line) (*p* = 0.012)

Observing the influence of MCTs, CD147 and GLUT1 expressions in CRC survival curves assessed by log-rank test, we found that positivity for MCT1 in the plasma membrane associated with better cumulative survival in CRC stage IV (*p* = 0.012) (Fig. 3), while no correlations were found for the remaining proteins (Table 9). The predictive prognostic value of MCT1 was analyzed by means of Cox proportional hazards regression model, however, multivariate analysis showed that only tumor differentiation remains as an independent factor with predictive value for overall survival (Table 10). No significant differences were found in the CRC lymph node and hepatic metastasis survival curves for the different proteins.

**Table 9:** Kaplan-Meyer survival curves *p* values

	Protein			
Stage	MCT1	MCT4	CD147	GLUT1
Stage I	0.427	0.627	0.639	0.162
Stage II	0.249	0.596	0.300	0.302
Stage III	0.958	0.157	0.526	0.733
Stage IV	0.012	0.253	0.434	0.604
Overall	0.722	0.317	0.503	0.285

**Table 10:** Prognostic factors for overall survival in CRC stage IV

	Overall survival					
Variable	Univariate analysis			Multivariate analysis		
	HR	95 % CI	*p*	HR	95 % CI	*p*
Age (<45 years)	2.116	0.938 – 4.774	0.071	0.898	0.271 - 2979	0.860
Localization (rectum)	0.684	0.350 – 1.447	0.267			
CEA (>5 ng/mL)	2.017	1.117 – 3.641	0.020	1.834	0.946 – 3.553	0.072
Differentiation (Poorly/undifferentiated)	2.748	1.470 – 5.138	0.002	3.488	1.563 – 7.782	0.002
Spread lymph node (present)	1.156	0.638 – 2.093	0.633			
Vessel invasion (present)	1.312	0.733 – 2.351	0.361			
MCT1 (+)	0.394	0.186 – 0.834	0.015	0.694	0.310 – 1.597	0.390
MCT4 (+)	1.429	0.767 – 2.664	0.261			
CD147 (+)	0.779	0.412 – 1.473	0.442			
GLUT1 (+)	1.169	0.642 – 2.129	0.610			

## Discussion

MCTs play an essential role in the maintenance of cancer glycolytic metabolism. On one hand, they perform the efflux of lactate and, on the other hand, they help in the regulation of the cell pH, by co-transporting a proton [8, 13–15, 17, 18]. Due to their upregulation in several cancers, they are currently seen as promising therapeutic targets [8, 12–18], with an inhibitor of MCT1 already in clinical trials (NCT01791595). Here we aimed to characterize the expression of MCT1, MCT4, CD147 and GLUT1 in a comprehensive series of CRC primary tumours, lymph node and hepatic metastasis, as well as to assess the clinical-pathological significance of their overexpression.

Our group has previously analyzed the immunoexpression of MCT isoforms 1, 2 and 4 in a series of 126 cases of CRC. Expression of all MCT isoforms in tumour cells was significantly increased, with a significant gain in membrane expression for MCT1 and MCT4 and loss for MCT2 in tumour cells, when compared to adjacent normal epithelium [32]. In the present study, we strengthen the previous results by increasing the number of primary CRC cases from 126 to 487 and also included 210 of lymph node metastasis of the same patients and 45 additional cases of CRC hepatic metastasis. We assessed the expression and the association between MCTs and additional proteins not previously studied (CD147 as MCT1/4 chaperone and the glycolytic protein marker GLUT1), to further understand the role of MCTs in the glycolytic metabolism remodeling of primary CRC and in metastasis.

Our results showed that most proteins studied (MCT4, CD147 and GLUT1) were overexpressed at the plasma membrane of CRC cells and CRC lymph node and hepatic metastasis when compared with CRC NA tissue, with exception of MCT1 in CRC lymph node and hepatic metastasis. Here we showed that in CRC samples, MCTs were the most frequently expressed proteins followed by CD147 and GLUT1. The MCT results are in concordance to our previous study, in which we showed upregulation of MCT1 and MCT4 in the tumour samples, compared to NA tissue [32]. We found that MCT1 expression was associated with CD147 in CRC primary samples and with GLUT1 in CRC hepatic metastasis. Expression of MCT4 was associated with CD147 and GLUT1 in all samples. It is known that the association of MCT1 and MCT4 with the cell surface glycoprotein CD147 is essential for their activity and proper expression at the plasma membrane [10, 48]. However, not always this association prevails in cancer tissue, suggesting the role of putative additional chaperones [9].

Most CRC cells, as many other solid tumours, rely mostly on glycolysis to meet their energetic demands [49]. Thus, the high rates of glucose uptake are accompanied by upregulation of glucose transporters. There are two types of sugar transporters in gut, facilitative Na + −independent sugar transporters (GLUT) and Na + −dependent sugar cotransporters (SGLT), which require energy for sugar transport. Increased expression of GLUT1 was described in various cancer tissues, including CRC, indicating that GLUT1 plays an important role in cancer and that its expression could be useful as a marker for malignant transformation [50–52]. Besides, overexpression of SGLT1 in CRC showed a correlation with higher clinical stages [53]. Our results showed association between MCT1 and MCT4 and GLUT1, supporting their role in glycolytic metabolism. To the best of our knowledge, this is the first report on this association in the context of CRC. Koukourakis group [31] described strong GLUT1 expression in CRC cells, although the association with MCTs was not assessed. It is likely that CRC cells upregulate GLUT1 to increase glucose uptake and the subsequent accumulated lactate is extruded by MCTs. Additionally, as far as we are aware, we show for the first time that the expression of MCTs, CD147 and GLUT1 are also present in CRC hepatic metastasis, suggesting the maintenance of this metabolic profile in the invasive phenotype.

To the best of our knowledge, this is the first report that compares the expression of these proteins in CRC primary tumour with the respective lymph node metastasis,. MCT1, CD147 and GLUT1 positivity were positively associated in CRC and lymph node metastasis, although the expression of MCT1 was less pronounced in the metastasis than the primary tumour, which suggests that metabolic profile of the lymph node metastasis may be different from the primary tumour. For MCT4, the maintenance of membrane expression in lymph node metastasis, suggests the predominance of glycolytic metabolism, but more studies are necessary to demonstrate this hypothesis. In studies performed in breast cancer, MCT expression is reduced in lymph node metastasis compared to primary tumour [54].

Lymph node metastasis are initially independent of vascularization, relying on the stroma to provide the required nutrients [54, 55]. It seems to exist a high expression of MCT4 in the tumour stroma and an association of this expression with a worse patient survival [55]. On the other hand, no association with prognosis was observed for epithelial MCT4 levels [55]. There is no data in the literature for none of the proteins studied in lymph node metastasis, so additional studies are necessary to confirm and explain this observation.

Regarding the association between the proteins under study in primary CRC and clinicopathological data, we found that MCT1 positivity was associated with older patients; CD147 was associated with both larger tumours and more advanced tumour stage. Our results are supported by previous observations showing CD147 might enhance CRC growth, thus being associated with poor clinical prognosis [56–58]. GLUT1 expression associated significantly with exophytic lesions, low CEA levels, poorly-differentiated tumours, and a tendency for association with the presence of lymph node metastasis. All of these features, with exception of low CEA levels, are characteristic of more aggressive tumours and poor prognosis. These associations support previous studies suggesting that GLUT1 may play an important role in tumour cell survival, by promoting an adequate energy supply [59, 60] and could be a useful biomarker for malignant transformation [50, 60].

Regarding the association between the protein expression in lymph node metastasis and the same clinicopathological data, MCT4 positivity was associated with colon tumours and more advanced tumour stage and CD147 with tumour differentiation. MCTs and CD147 work synergistically, increasing invasiveness and metastatic potential trough microenvironment acidification and extracelular matrix destruction, via metalloproteinase induction [61–63]. Studies with growth factors and metalloproteinases in lymph nodes reveal expression similar to the primary tumour, suggesting that primary tumours acquire an invasive phenotype and that these characteristics are maintained in the metastasis [61]. For CD147, we were unable to show that lower tumor differentiation corresponds to higher membrane expression, as observed in other studies [51, 64], but our sample of poorly and undifferentiated tumours was small (*n* = 16 and n = 1, respectively), which may have compromised statistical power.

Data on associations between protein expression in hepatic metastasis with the clinicopathological revealed that MCT1 expression was associated with primary tumour localization in colon. Association with left colon is a poor prognosis factor since CRC located in the left colon is associated with worse prognosis [65].

Analyzing the CRC survival curves, we observed that MCT1 plasma membrane expression was associated with better patient survival in stage IV, however this association was not confirmed by multivariate analysis. MCT1 plays a pivotal role in colon epithelial cell metabolism, being critical for the metabolic communication between cells and for the transport of short chain fatty acids (SCFA), including lactate [29, 66, 67]. Indeed, gut microbial-derived SCFA, namely acetate, propionate and butyrate, exert multiple beneficial effects on the colon energy metabolism [66–69]. SCFA were demonstrated “in vitro” and “in vivo” to induce apoptosis of CRC cells but not of normal colon cells, protecting normal colon mucosa [70, 71]. Our group has recently demonstrated that acetate induces lysosomal membrane permeabilisation and the release of Cathepsin D [70]. In this sense, overexpression of MCT1 will increase not only the uptake of SCFA but also the transport of lactate into the CRC cells inducing intracellular acidification [17], and consequently will potentiate CRC cells apoptosis.

No significant differences were found in primary tumour, CRC lymph node and hepatic metastasis survival curves for the different proteins.

## Conclusions

Overall, our findings support the role of MCT1, MCT4, CD147 and GLUT1 in CRC maintenance and progression. Moreover, since we found upregulation of these molecules either in primary tumours or metastasis, our results also support their exploitation as molecular targets in CRC treatment.

## Abbreviations

CEA, Carcinoembryonic antigen; CRC, Colorectal cancer; MCTs, Monocarboxylate transporters; MSI, Microsatellite Instability; MSI-H, High MSI; MSI-L, Low MSI; MSS, Microsatellite stable; NA, Normal adjacent epithelium; SCFA, Short chain fatty acids; SMCTs, Sodium-coupled monocarboxylate co-transporters; TMAs, Tissue microarrays
